# El programa del manejo integrado de vectores en el marco de la pandemia por COVID-19 en Medellín, Colombia

**DOI:** 10.7705/biomedica.6679

**Published:** 2023-03-30

**Authors:** Raúl A. Rojo-Ospina, Marcela Quimbayo-Forero, Arley Calle-Tobón, Sindy C. Bedoya-Patiño, Maribel Gómez, Astrid Ramírez, Johnny Sánchez, Juan F. Silva- Alzate, Carlos J. Montes-Zuluaga, Jorge M. Cadavid, Enrique A. Henao-Correa

**Affiliations:** 1 Programa de Control de Vectores, Secretaría de Salud, Alcaldía de Medellín, Medellín, Colombia Secretaría de Salud Medellín Colombia; 2 Grupo Entomología Médica, Universidad de Antioquia, Medellín, Colombia Universidad de Antioquia Universidad de Antioquia Medellín Colombia

**Keywords:** Aedes, dengue, enfermedades transmitidas por vectores, COVID-19, arbovirus, Aedes, dengue, COVID-19, vector borne diseases, arbovirus

## Abstract

**Introducción.:**

La pandemia por COVID-19 presionó los sistemas de salud para mantener alerta y activos los programas de control y prevención de las enfermedades transmitidas por vectores, y generó cambios en las estrategias de control vectorial en áreas urbanas afectadas por el dengue, el Zika y el chikunguña.

**Objetivo.:**

Describir las adaptaciones del programa de vigilancia y control de vectores en Medellín durante la contingencia sanitaria por COVID-19.

**Materiales y métodos.:**

Iniciada la emergencia sanitaria, se elaboraron protocolos de bioseguridad. Se fortaleció la vigilancia entomológica institucional en lugar de las viviendas. La información se recolectó en Medellín durante los años 2018 a 2021, en las actividades del programa de vigilancia y control de vectores, que incluyen la vigilancia epidemiológica y entomo-virológica, el levantamiento de los índices entomológicos, el monitoreo de ovitrampas, la movilización social y comunitaria, la búsqueda y eliminación de criaderos, y el control químico; estas acciones se adaptarons o incrementaron para favorecer, de una parte, el autocuidado de las comunidades en confinamiento total y parcial, y de desarrollar las acciones de prevención y control.

**Resultados.:**

Se incrementó en un 40 % la vigilancia del mosquito mediante ovitrampas, la vigilancia entomo-virológica presentó un incremento de 34,4 % en el 2020 respecto al 2019, y se utilizaron herramientas virtuales para mantener y mejorar el contacto con la comunidad.

**Conclusión.:**

La pandemia por COVID-19 causó gran impacto en los programas de prevención y control de las enfermedades transmitidas por vectores. Medellín adaptó rápidamente las actividades de vigilancia entomo-virológica, las acciones de control y la comunicación con la comunidad durante la pandemia, y esto permitió mantener activo el programa del manejo integrado de vectores en la ciudad.

## Abstract (en)

**Introduction:**

The COVID-19 pandemic pressured health care systems to remain alert and active in their vector-borne disease control and prevention programs, leading to changes in vector control strategies in urban areas affected by dengue, Zika and chikungunya.

**Objective:**

To describe the adaptations made to the vector control and surveillance program in Medellín during the COVID-19 health emergency.

**Materials and methods:**

Once the health emergency started, biosecurity protocols were developed. Entomological surveillance was strengthened from the institutional environment instead of homes. Data was collected in Medellín from 2018 to 2021 during the vector control and surveillance program activities, which included epidemiological and entomo- virological surveillance, entomological index survey, ovitrap monitoring, community mobilization, search and elimination of mosquito breading sites, and chemical control.

These actions were adapted and/or increased to promote self-care among communities in total and partial confinement, and to develop prevention and control measures.

**Results:**

Mosquito monitoring was increased by 40% using ovitraps, entomological- virological surveillance showed an increase in 2020 of 34,4% compared to 2019 and virtual media was used to keep and improve contact with the community.

**Conclusion:**

The COVID-19 pandemic had a significant impact on arbovirus prevention and control programs. The city of Medellín quickly adapted its entomo-virological surveillance activities, control measures, and the contact with the community during the pandemic, which allow the Integrated Vector Management program to remain active in the city.

El síndrome respiratorio agudo grave por COVID-19 fue identificado y reportado por primera vez en Wuhan, China, en el año 2019, siendo el virus SARS-CoV-2 (Coronaviridae) su agente etiológico ^1^. La enfermedad se propagó de forma exponencial, generando rápidamente una amenaza global, por lo cual, la Organización Mundial de la Salud (OMS) la declaró pandemia en marzo de 2020 y, en Colombia, se hizo la declaración el 12 de marzo de 2020 ^2^. Esta emergencia sanitaria obligó a los países de Latinoamérica a enfrentar dos desafíos de riesgo simultáneo, la concurrencia de la pandemia desencadenada por la COVID-19 y las enfermedades infecciosas preexistentes en la región transmitidas por vectores de alto impacto en la salud pública, como dengue, Zika y chikunguña ^3^.

Las enfermedades causadas por arbovirus, como el dengue, para las cuales aún no existe vacuna o medicamentos, son controladas desde el enfoque ambiental, con el apoyo de información epidemiológica, dirigiendo los esfuerzos a la estrategia de manejo integrado de vectores como la herramienta disponible y efectiva para evitar la transmisión de estas enfermedades ^4^.

Diferentes directrices fueron publicadas en el año 2020 por la OMS en conjunto con la Organización Panamericana de la salud (OPS), que pretendieron unificar criterios y brindar directrices para mantener y adaptar los programas del manejo vectorial en el escenario de la pandemia:

- estratificación de dengue;
- caracterización de los criaderos de mosquitos vectores;
- manejo de los criaderos más frecuentes;
- implementación de control del vector adulto, y
- cuidado y protección del agente de salud durante la visita domiciliaria. Estas directrices favorecieron la continuidad adecuada del control de las enfermedades transmitidas por vectores ^5^^-^^7^.

Para el caso de Colombia, estas directrices fueron adoptadas e impartidas por el Ministerio de Salud y Protección Social, que pretendió establecer las orientaciones generales para la operación de los equipos territoriales encargados del manejo de las enfermedades transmitidas por vectores, garantizando la continuidad de las acciones necesarias de promoción, prevención y control de enfermedades como el dengue y la malaria a nivel nacional, departamental y municipal, en el marco de la pandemia por COVID-19 ^8^^,^^9^.

En este trabajo, se describen los retos, las adaptaciones y la intensificación de las acciones del programa de vigilancia y control de vectores de Medellín (Colombia), escenario histórico de circulación viral para dengue, Zika y chikunguña, ante la contingencia sanitaria desencadenada por la COVID-19.

## Materiales y métodos

### Área de estudio

La ciudad de Medellín se encuentra situada a 75° 34' 05" LO y 6° 13' 55" LN, tiene una extensión de 376,2 km^2^ y es atravesada de sur a norte por el río Aburrá-Medellín. La topografía de la ciudad es un plano inclinado que desciende desde los 1.800 a los 1.500 m.s.n.m, con clima subtropical subhúmedo y una temperatura en la zona urbana que oscila entre los 16 y los 28 °C ^10^. Según el censo del DANE 2018, la población es de 2’.427.129 habitantes ^11^. La ciudad se encuentra dividida en 21 comunas, con circulación de los virus del dengue, del Zika y de chikunguña en 19 de ellas.

Las actividades del programa de control del dengue en Medellín se han diseñado bajo las cinco líneas claves de la estrategia de manejo integrado de vectores de la OPS ^12^.

Estas actividades se fundamentan en la información obtenida de los datos epidemiológicos, e incluyen el levantamiento de índices entomológicos, vigilancia de ovitrampas, vigilancia entomo-virológica, inspección de factores de riesgo, y movilización social y comunitaria que, de manera integral, permiten la planificación de las acciones de promoción, prevención y control.

## Actividades de vigilancia

### Datos epidemiológicos

La vigilancia epidemiológica para el seguimiento de los casos de las enfermedades transmitidas por vectores se realiza de manera permanente mediante la notificación en el Sistema Nacional de Vigilancia en Salud Pública (SIVIGILA), implementado en el 100 % de las instituciones prestadoras de servicios de salud encargadas de garantizar la atención oportuna a las personas y su notificación. La información obtenida de este sistema fue analizada para la identificación de brotes, la caracterización de la población en riesgo, la tendencia del evento, la identificación de alertas epidemiológicas y la determinación de los sitios por intervenir con acciones de control.

### Levantamiento de índices entomológicos

Esta actividad tiene por objeto cuantificar el riesgo entomológico de enfermar por alguna de las arbovirosis transmitidas en la ciudad. Para ello, se hicieron encuestas en viviendas, establecimientos educativos (primaria, secundaria, tecnológicos y universitarios) y establecimientos de salud. Su objetivo fue obtener datos de los potenciales criaderos, los criaderos positivos (presencia de larvas), los establecimientos positivos (con criaderos positivos) y la presencia de mosquitos adultos. Esta información fue utilizada para obtener los índices de Breteau, de depósitos, de adultos y de viviendas ^6^.

A partir de la declaratoria de emergencia sanitaria por la pandemia por COVID-19 (marzo del 2020) y ante la imposibilidad de realizar actividades de levantamiento entomológico dentro de las viviendas en Medellín, la Secretaría de Salud realizó los siguientes ajustes en el desarrollo de esta actividad:

- suspensión de las actividades de levantamiento de índices entomológicos dentro de las viviendas y en los establecimientos de salud, y
- fortalecimiento de las actividades de levantamiento de índices a partir de la red de establecimientos educativos de la ciudad y la utilización de estos como sensores de riesgo entomológico comunitario.

La modificación planteada para llevar a cabo el levantamiento entomológico a nivel institucional tuvo por objeto usar la información obtenida a nivel institucional como sensor de los territorios vigilados, y generar alertas para la identificación y el manejo oportuno de depósitos crípticos y comunes a nivel institucional (tanques de almacenamiento, cunetas, canoas, aparatos sanitarios, material en desuso y sumideros, entre otros). Una vez el gobierno nacional levantó las medidas de confinamiento, se retomaron gradualmente las actividades de levantamiento de índices dentro de las viviendas (septiembre de 2020).

## Actividades de vigilancia entomológica

### Monitoreo de ovitrampas

Las ovitrampas están constituidas por una tarrina de color negro con capacidad de 2 litros, a la cual se adiciona un litro de agua limpia y se sumerge una tablilla de balso de 22 x 4x 0,2 cm y se ubica formando un ángulo de 45°, en la cual ovipositan las hembras de *Aedes* (*Stegomyia*) *aegypti* (Linnaeus, 1762) y las de *Aedes (Stegomyia*) *albopictus* (Skuse, 1894). Las tablillas se recogieron semanalmente y se llevaron al insectario para el conteo de huevos; las tablillas positivas, con presencia de huevos, se sumergieron en agua para obtener los adultos y hacer la identificación de especie.

### Vigilancia entomo-virológica

La vigilancia entomo-virológica tiene como objetivo detectar oportunamente la infección natural con los virus del dengue, del Zika o de chikunguña, en mosquitos recolectados en campo. Para obtener las muestras, se hicieron capturas utilizando una red entomológica y aspiradores bucales, en los diferentes espacios de las instituciones. Los mosquitos fueron transportados vivos al insectario, donde se identificaron mediante claves morfológicas ^13^. Se seleccionaron los especímenes de *A. aegypti A. albopictus y Culex quinquefasciatus* (Say, 1826) (Diptera:Culicidae) para detectar los virus del dengue ,del Zika y chikunguña, empleando el protocolo previamente descrito ^14^^-^^16^.

## Actividades de promoción, prevención y control

### Estrategia de movilización social y comunitaria

Esta estrategia fue empleada para crear mecanismos de comunicación por medio del fortalecimiento de grupos sociales mediante la movilización comunitaria, en la que se impartieron instrucciones que conducen al desarrollo de acciones de promoción y prevención de enfermedades transmitidas por vectores. Durante la emergencia sanitaria por COVID-19, el contacto con la comunidad se llevó a cabo mediante el uso de plataformas virtuales como WhatsApp, Zoom, Google Meet y Microsoft Teams. Mediante este mecanismo, se pudo mantener la comunicación y realizar encuentros sincrónicos virtuales con líderes comunitarios y grupos de estudiantes de educación básica y media de instituciones educativas de Medellín, que hacen parte de la estrategia de comités estudiantiles antidengue.

El desarrollo de las acciones de movilización social conjugó la epidemiología y las condiciones socioeconómicas de la ciudad, y fundamentó su trabajo en:

- programar capacitaciones virtuales y jornadas especiales de sensibilización en torno al conocimiento y la prevención de las enfermedades transmitidas por vectores;
- mantener y actualizar las bases de datos y el contacto permanente con las organizaciones involucradas en los procesos de información, educación y comunicación;
- realizar trabajo conjunto con instituciones aliadas que permitieron construir mapas de riesgo para priorizar acciones de control de enfermedades transmitidas por vectores;
- diseñar piezas gráficas físicas y digitales con mensajes educativos, que permitan a la comunidad identificar, prevenir, eliminar o controlar los factores de riesgo inherentes a las enfermedades transmitidas por vectores, y
- hacer encuestas virtuales empleando las redes sociales de la Alcaldía de Medellín.

### Búsqueda y eliminación de criaderos

Debido a la contingencia sanitaria, esta actividad en las viviendas fue adaptada a la estrategia de búsqueda y eliminación de criaderos durante la pandemia de COVID-19 con enfoque educativo, sin ingresar a las viviendas. La actividad se enfocó en que el funcionario orientara desde el exterior a los miembros del hogar para identificar y eliminar los posibles criaderos de mosquitos, desarrollar prácticas preventivas, y reconocer la enfermedad, sus síntomas y sus señales de alarma. La estrategia de búsqueda y eliminación de criaderos se llevó a cabo, principalmente, en viviendas y establecimientos educativos, de salud o comerciales.

### Control químico

Siguiendo las directrices del Ministerio de Salud, la aspersión de insecticidas se realizó con el objeto de reducir la población de mosquitos en aquellos lugares donde se presentaron casos reportados en el SIVIGILA, se detectaron virus en los análisis entomo-virológicos, o gran productividad, positividad o ambas en las ovitrampas, y en última instancia, por los resultados de los índices entomológicos.

En el desarrollo de las acciones de control químico, se emplearon dos técnicas:

- Aspersión de insecticida (Malatión®) con máquinas termonebulizadoras dentro de las viviendas, los establecimientos educativos de primaria, secundaria, universitarios o tecnológicos, y en áreas comerciales.
- Aspersión de insecticida (Malatión®) con máquina ULV (*Ultra Low Volume*) montada en un vehículo en vías públicas de fácil circulación.

## Resultados

El desarrollo de la emergencia sanitaria por COVID-19 conllevó, en primera instancia, la elaboración y aprobación de protocolos de bioseguridad por parte del operador del programa, ajustados a las especificaciones trazadas desde el Ministerio de Salud y Protección Social de Colombia, y al cumplimiento de las directrices formuladas desde esta misma instancia para el desarrollo de las acciones del programa de control de vectores.

### Situación epidemiológica

**Figura 1:**
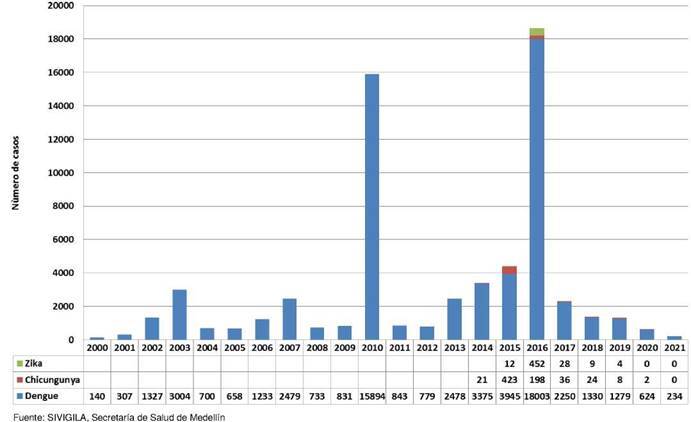
Número de casos de dengue, Zika y chikunguña en Medellin, Colombia, entre el 2000 y el 2021

Durante los últimos cinco años, en Medellín, se presentó una disminución constante en el número de casos de arbovirosis; particularmente, el 2021 fue el periodo con menor número de casos reportados en los últimos 20 años (figura 1), distribuidos en todas las comunas de Medellín.

Fuente: SIVIGILA, Secretaría de Salud de Medellín

En la emergencia sanitaria derivada por la pandemia de COVID-19, las autoridades nacionales de salud no realizaron ninguna modificación ni ajustes a la captación, búsqueda y notificación de eventos relacionados con las enfermedades transmitidas por vectores.

### Levantamiento de índices entomológicos

**Figura 2:**
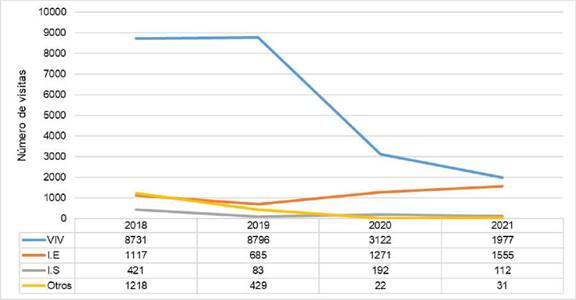
Tipo y cantidad de predios inspeccionados para el levantamiento de índices entomológicos entre el 2018 y el 2021 en Medellín

VIV: viviendas; IE: instituciones educativas de primaria, secundaria, tecnológicas y universitarias; IS: instituciones de salud; Otros: áreas comerciales, parques, construcciones, etc.

Durante el 2018 y el 2019, años prepandémicos, se realizaron 11.487 y 9.993 visitas para levantamientos de índices entomológicos en la ciudad, respectivamente. El 81,5 % de las visitas fueron realizadas en viviendas, el 8,4 % en instituciones educativas, el 2,3 % en instituciones de salud y el restante en otro tipo de predios, como áreas comerciales, parques públicos y construcciones (figura 2). Durante el 2020, se presentó una disminución en las visitas realizadas, para un total de 4.607; el 67,7 % fueron inspecciones en viviendas, mientras que las visitas en instituciones educativas correspondieron al 26,6 %. En 2021, las visitas a viviendas e instituciones educativas fueron del 52,4 % y el 42,3 %, respectivamente, de un total de 3.675 visitas durante ese año.

### Monitoreo de ovitrampas

Durante los años 2018 y 2019, la red de monitoreo de ovitrampas para la vigilancia de las poblaciones de *A. aegyptiy A. albopictus* en Medellín estaba constituida por 250 trampas distribuidas uniformemente en la ciudad, las cuales estaban ubicadas en viviendas, instituciones educativas, instituciones de salud y otros (cuadro 1). Al momento de la declaración de emergencia por COVID-19, ante la imposibilidad de realizar actividades entomológicas en las viviendas y con el propósito de fortalecer el sistema de vigilancia, se hizo una redistribución de las ovitrampas ante el cierre de algunos establecimientos educativos y se incrementó la red a 352 (incremento del 40,8 %). Se contó con el apoyo de la Secretaría de Educación y algunos establecimientos de educación privada que garantizaron el acceso de los funcionarios para hacer el seguimiento.

**Cuadro 1:** Número de ovitrampas activas entre 2018 y 2021 por tipo de predio en Medellín, Colombia

Tipo de establecimiento	2018	2019	2020				**2021**
			Semestre 1	Desinstaladas por COVID	Semestre 2	Desinstaladas por COVID	
Viviendas	12	14	0	0	0	0	0
Instituciones educativas de primaria y secundaria	194	187	210	11	313	0	312
Instituciones de salud	8	9	12	0	13	1	13
Tecnológicos y universidades	10	10	10	2	13	0	12
Otros	26	30	18	3	13	0	15
Total	250	250	250	16	352	1	352

Otros: áreas comerciales, parques, construcciones, etc.

**Figura 3:**
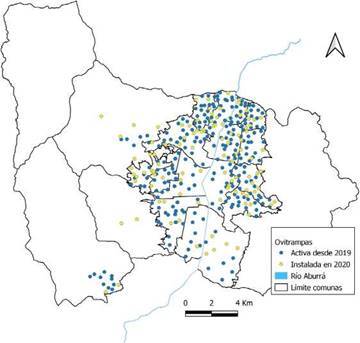
Distribución del sistema de ovitrampas en Medellin. En azul se muestran las ovitrampas activas desde 2019 y, en amarillo, las ovitrampas instaladas como adaptación a la emergencia sanitaria por COVID-19.

La vigilancia con ovitrampas mantuvo la línea base establecida en años anteriores, y focalizó las nuevas ovitrampas para cubrir y densificar todo el territorio municipal, con mayor concentración de estas donde históricamente el comportamiento de las arbovirosis ha presentado una mayor incidencia (figura 3).

### Vigilancia virológica

El procesamiento de muestras de mosquitos para detectar los virus del dengue, del Zika y del chikunguña, hace parte de la vigilancia rutinaria del programa de manejo integrado de vectores de Medellín. Durante el 2018, se procesaron 1.579 *pools* de mosquitos, de los cuales el 9 % resultó positivo para la presencia de alguno de estos arbovirus (cuadro 2). El 87,7 % de los *pools* positivos fueron de *A. aegypti*. El virus del Zika fue el de mayor circulación en este periodo (54,4 %), principalmente en *A. aegypti',* pero también, se encontró en *A. albopictus y C. quinquefasclatus*. Durante el 2019, se procesaron 1.163 *pools*: se encontró 5,8 % de positividad y el virus del dengue fue el más abundante, registrándose en las tres especies de mosquitos evaluados (cuadro 2).

**Cuadro 2:** Cantidad de pools de mosquitos evaluados para la detección de los virus del dengue, del Zika y el chikunguña entre los años 2018 y 2021 en Medellín, Colombia

Año	Pools procesados			Pools positivos								
	*A. aegypti*	*A. albopictus*	*C. quinquefasciatus*	*A. aegypti*			*A. albopictus*			*C. quinquefasciatus*		
				DENV	ZIKV	CHIKV	DENV	ZIKV	CHIKV	DENV	ZIKV	CHIKV
2018	1298	88	193	49	80	0	5	4	0	0	6	3
2019	940	90	133	11	1	1	2	1	0	1	0	1
2020	1207	222	134	109	3	3	12	0	0	7	0	0
2021	954	256	211	10	0	0	2	2	0	1	0	0

Debido a la emergencia sanitaria por COVID-19 en el 2020, se suspendieron las capturas dentro de las viviendas y se concentró la captura de mosquitos en establecimientos educativos; esto permitió conformar y evaluar 1.563 *pools*, lo que reflejó un incremento del 34,4 % de muestras analizadas respecto al año inmediatamente anterior y arrojó una positividad del 8,3 %. En este año, se registró una gran circulación del virus del dengue, con registros en las tres especies de mosquitos, mientras que, del Zika y del chikunguña se tuvieron tres *pools* de *A. aegypti* positivos. En el año 2021, se registró la positividad más baja del periodo de estudio: del 1,1 % en un total de 1.421 *pools* de mosquitos evaluados. En este periodo, se encontró circulación del virus del dengue en las tres especies de mosquitos y, del virus del Zika, sólo en *A. albopictus* (cuadro 2).

### Estrategias de control

#### Búsqueda y eliminación de criaderos

Esta actividad se llevó a cabo principalmente en las viviendas. En el 2018 y el 2019, se inspeccionaron cada año más de 40.000 viviendas (cuadro 3). Sin embargo, durante el 2020, esta actividad se realizó en 16.839 viviendas, las cuales fueron visitadas durante el primer y el último trimestre de ese año. Mediante la estrategia de búsqueda y eliminación de criaderos durante la pandemia de COVID-19, se realizó la capacitación en 1.248 viviendas durante el 2020, la mayoría de estas durante el segundo y el tercer trimestre del año. Esta actividad continuó implementándose durante el 2021, lográndose en ese año capacitar los habitantes de 1.090 hogares, aunque ya el ingreso a las viviendas estaba permitido.

**Cuadro 3:** Número y tipo de predios inspeccionados para la búsqueda y eliminación de criaderos entre 2018 y 2021 en Medellín, Colombia

Año	VIV	IE	IS	Otros	BEC bajo Covid-19
2018	43.499	65	8	604	0
2019	41.379	320	65	734	0
2020	16.839	426	68	142	1.248
2021*	6.872	204	30	47	1.090

VIV: viviendas; IE: instituciones educativas de primaria, secundaria, tecnológicas y universitarias; IS: instituciones de salud; Otros: áreas comerciales, parques, construcciones, etc.

* Interrupción contractual de actividades para el periodo del segundo semestre de 2021.

#### Control químico

La ejecución de actividades de control químico en el 2018 y el 2019, no presentó diferencias significativas en el número de predios intervenidos, con 36.923 y 37.303, respectivamente. Durante el 2020, bajo la contingencia por COVID-19, esta actividad presentó un incremento del 65 % en la cantidad de predios en comparación con los dos años anteriores, por lo cual se hizo control químico en 61.578 predios. Este incremento obedeció a la contabilización de la actividad tomando viviendas, locales comerciales o ambos, ubicados en el primer piso en las que abrieron las puertas y ventanas para el ingreso del insecticida mediante la aspersión desde el exterior, en las áreas señaladas de control. En el 2021, la cantidad de predios fumigados fue 3.622, una cantidad 17 veces menor en comparación con el 2020, debido a la interrupción de los procesos contractuales, la escasa circulación de arbovirus en mosquitos (cuadro 2) y la baja tasa de incidencia de la enfermedad (figura 1).

#### Movilización social y comunitaria

Las actividades de movilización social que se realizaron de forma presencial, como reuniones, charlas educativas, conversatorios y capacitaciones en los años 2018 y 2019, permitieron sensibilizar 12.800 y 15.750 personas, respectivamente, en torno a la biología, transmisibilidad y epidemiología de enfermedades transmitidas por vectores; en cambio, en el primer trimestre de 2020, se sensibilizaron 4.807 personas. Después de decretada la emergencia por COVID-19 en Colombia, a partir de julio del 2020, se implementaron estrategias de comunicación virtual con población docente y estudiantil mediante el uso de plataformas digitales. Se concertó, planeó y coordinó con cinco instituciones educativas de la ciudad, con el propósito de reactivar los comités estudiantiles antidengue. Finalmente, esto permitió, llevar a cabo 56 encuentros sincrónicos con 28 grupos estudiantiles en los que participaron alrededor de 1.100 escolares, de los grados 3° a 11°. Así, se desarrollaron experiencias dirigidas virtualmente que permitieron abordar temas sobre el conocimiento de la enfermedad y actividades de campo en los lugares de residencia.

Además, se estableció comunicación telefónica con organizaciones de base y líderes comunitarios, con el propósito de difundir información relevante respecto al dengue y las especies vectoras, y hacer la coordinación social para desarrollar acciones en el territorio.

De otra parte, se logró implementar una encuesta virtual a través de las redes sociales de la Alcaldía de Medellín, la cual permitió la participación de 925 personas que manifestaron la presencia o ausencia de mosquitos en sus viviendas, arrojando como resultado que las comunas de Belén, Laureles, Buenos Aires y Robledo tenían una mayor proliferación de mosquitos.

## Discusión

Colombia es uno de los países de Latinoamérica con mayor incidencia de enfermedades transmitidas por vectores ^17^. Dentro del país, Medellín ha sido una de las ciudades que aporta un importante número de casos de arbovirosis, particularmente de dengue, con picos epidémicos en los años 1998, 2003, 2007, 2010 y 2016 (figura 1); estos dos últimos se destacan como los años de mayor incidencia, con cerca de 18.000 casos reportados ^18^. Este comportamiento ha sido promovido por condiciones ambientales y socioculturales que propician una importante presencia de *A. aegypti* en todos los barrios de la ciudad ^19^. Además, se suma la presencia del mosquito *A. albopictus* que fue reportado en el 2011 ^20^, especie que ha sido encontrada infectada naturalmente con los virus del dengue y del Zika ^14^^,^^16^^,^^21^. A partir del 2017, la incidencia de dengue en Medellín ha presentado una continua disminución, posiblemente favorecida por el fortalecimiento de las actividades de vigilancia y prevención orientadas por el programa del manejo integrado de vectores.

Entre el 2019 y principios de 2020, Colombia afrontó una epidemia de dengue; sin embargo, tras la declaratoria de la emergencia sanitaria por COVID-19, se presentó un rápido descenso en el número de casos reportados ^3^. Medellín no fue la excepción pues, según el comportamiento epidemiológico del dengue observado a partir del año 2020, su número ha sido el más bajo en los últimos 20 años y se ha mantenido una tendencia al descenso en los reportes de casos.

En algunas regiones se reportó un comportamiento similar, y se ha planteado la discusión sobre si el descenso de casos correspondió a una disminución en la incidencia del dengue *per se* o a un subregistro de casos ^22^^,^^23^. Para comprender ese comportamiento epidemiológico de descenso en el número de casos, se deben considerar diversos factores involucrados, como lo es el confinamiento de las personas por la contingencia de la COVID-19, el cual redujo la movilidad de las personas y, por ende, la circulación viral ^24^.

No obstante, la posible infestación del vector dentro de las viviendas, que podría aumentar la exposición y el riesgo de las personas, se controló mediante un incremento en las acciones de control químico en las viviendas, como se detalla más adelante. Por otra parte, están factores como la prioridad del diagnóstico de COVID-19, el encarecimiento de los insumos para las pruebas diagnósticas, la concentración de actividades de atención personalizada en las instituciones de salud para los casos de COVID-19, el temor de las personas para acudir a centros asistenciales a consultar, y la congestión en las líneas de atención, entre otros, se suman al impacto directo e indirecto en la captación y el reporte de casos. Ante esta situación, la OPS estableció un conjunto de recomendaciones para continuar y adaptar los programas del manejo integrado de vectores en la región, los cuales se tuvieron en cuenta para llevar a cabo las acciones de control ^25^.

Ante el inicio de las medidas de confinamiento y distanciamiento social el 25 de marzo de 2020 en Colombia, la Secretaría de Salud de Medellín ajustó su programa de manejo integrado de vectores para continuar con las campañas de vigilancia, prevención y control, para acoplarse a las nuevas condiciones de operatividad que le permitieron dar continuidad al desarrollo de las acciones y a la disminución constante de los factores de riesgo. Para esto, se suspendieron las actividades de vigilancia y control dentro de las viviendas e instituciones de salud, y se incrementó el número de actividades en establecimientos educativos, a los cuales no estaban asistiendo los estudiantes.

Para mejorar la vigilancia de las densidades poblacionales de mosquitos, se incrementó en un 41 el número de ovitrampas en la ciudad y se incrementó el número de muestras obtenidas mediante levantamientos de índices. Las ovitrampas se utilizaron para el análisis entomo-virológico, con un aumento del 34 % en comparación con el 2019; se lograron identificar 134 puntos en la ciudad con circulación de arbovirus en mosquitos, lo cual permitió focalizar las acciones de control.

Cabe resaltar que nuestra experiencia operativa de adaptación en las acciones de control y reajuste del manejo integrado de vectores, se suma a los trabajos realizados en otras ciudades, como Yogyakarta en Indonesia, y en Malasia, los cuales modificaron y ajustaron las medidas de control y prevención de vectores durante la pandemia, asumiendo retos similares en cuanto a la planeación, modificación y reajuste de actividades de fumigación, distribución de ovitrampas y acoplamiento de actividades comunitarias ^26^^,^^27^.

Durante el 2020, se registró un incremento en el número de muestras positivas para dengue, en comparación con los años 2018 y 2019, lo cual sugiere que en este periodo se presentó un aumento en la circulación de esta enfermedad en Medellín; sin embargo, las condiciones derivadas de la emergencia sanitaria limitaron la capacidad de los sistemas de salud para identificar los casos ^28^.

Con el objetivo de reducir la circulación viral en mosquitos que pudiera darse a partir de la aparición de nuevos casos en la ciudad o por la obtención de resultados positivos en las muestras para análisis virológico de los mosquitos capturados en campo, la Secretaría de Salud, atendiendo la directriz impartida desde el Ministerio de Salud ^8^, durante 2020 intensificó el control químico en la ciudad en 60,5 % con respecto al 2019, esto permitió cortar o reducir el avance de la enfermedad.

Es importante resaltar que las actividades de control químico con termonebilizadoras realizadas durante el primer trimestre de 2020, se llevaron a cabo en el interior de las viviendas, en las zonas de intervención que cumplían los criterios según los lineamientos del programa establecidos por el Ministerio de Salud, además de la positividad de los resultados virológicos en mosquitos y la determinación de riesgo entomológico por ovitrampas, las demás intervenciones realizadas en el resto del año 2020 y en el 2021 se efectuaron desde el exterior de las viviendas y se contabilizaban todas aquellas (primeros y segundos pisos) que abrían sus puertas y ventanas. De igual manera, es importante destacar que durante el año 2021 el programa no operó durante varios meses debido a interrupciones en los procesos contractuales.

El temor al contagio por COVID-19 de los moradores de las viviendas de los sectores priorizados de intervención, sumado a la restricción de ingreso a las mismas, fueron determinantes para realizar cambios en la estrategia de búsqueda y eliminación de criaderos al interior de las viviendas. Esta situación se reflejó finalmente en una reducción del 60,3 % de las actividades ejecutadas durante el año 2019, lo cual llevó a enfocar la realización de la actividad, respetando la distancia de seguridad con las personas, a suprimir la entrega de material educativo y a implementar estrategias de comunicación asertiva en los encuentros voz a voz con el propósito de brindar información sobre la enfermedad, sus síntomas, señales de alarma, sitios de reproducción y las medidas de prevención al interior de las edificaciones, entre otros.

Las acciones de movilización social que se desarrollaban de forma presencial, telefónica y por medio de canales digitales previo a la pandemia, se fortalecieron con el diseño e implementación de una metodología virtual que permitiera acercarnos a la comunidad y especialmente dar continuidad con la estrategia de los comités estudiantiles antidengue en las instituciones educativas, para difundir el mensaje de prevención de las enfermedades transmitidas por vectores. Experiencias similares se han realizado en otros programas de control y proyectos de investigación en localidades como Tolima (Colombia) ^29^ y Buenos Aires (Argentina) ^30^.

La estrategia tuvo como propósito mantener un acercamiento y una comunicación directa con grupos de estudiantes, principalmente, para movilizarlos en torno a la prevención y control del dengue; además, que se llevara a la práctica dentro de sus entornos familiares lo aprendido durante los encuentros virtuales sincrónicos. Esta estrategia virtual se convirtió en una prueba piloto que funcionó como alternativa para informar, comunicar y mejorar los conocimientos sobre la transmisión de dichas enfermedades, y propiciar la corresponsabilidad de los estudiantes y sus familias en el control de los factores de riesgo asociados a las arbovirosis. No obstante, hubo dificultades a la hora de hacer seguimiento a las acciones prácticas de los estudiantes dentro de sus entornos y viviendas.

Por otro lado, con las publicaciones de piezas gráficas en las redes sociales de la Alcaldía de Medellín y su difusión por medio de estados, grupos de WhatsApp y correos electrónicos institucionales, se logró dar mayor alcance a la campaña de prevención del dengue, llegando a cientos de personas directa e indirectamente. Sin duda, el uso de herramientas digitales como las redes sociales llega a una gran cantidad de población que se conecta con frecuencia a la virtualidad; sin embargo, resulta necesario hacer análisis más precisos para evaluar cuál es el impacto a mediano plazo sobre las estrategias de movilización social virtual y el uso piezas gráficas para identificar los ajustes para la implementación de esta metodología.

### Desafíos y limitaciones de estudios

Durante el desarrollo del trabajo bajo contingencia, el primer obstáculo que enfrentamos fue la seguridad de los trabajadores que ejecutaban labores en campo. Sin embargo, la solución inmediata fue dotarlos con los elementos de protección personal sugeridos por las autoridades de salud. Además, se hicieron capacitaciones sobre el uso de estos elementos, y la seguridad biológica y psicológica brindada por las aseguradora de riesgos laborales.

Un obstáculo que se presentó se debió a la priorización de las vacunas exclusivamente para el personal asistencial en salud (médicos, enfermeras, laboratoristas, etc.); asimismo, hubo vigilancia permanente ante cualquier contacto cercano y manifestación de síntomas. Otros desafíos fueron la adaptación rápida de las actividades de control, el cambio de disposición de ovitrampas en viviendas a instituciones educativas, y la modificación de la estrategia de búsqueda y eliminación de criaderos intradomiciliaria a una forma educativa. Finalmente, no hubo cambios significativos en cuanto a la disposición o limitación de recursos financieros dirigidos para desempeñar las acciones de control.

En conclusión, la Secretaría de Salud de Medellín logró rápidamente adaptar y mantener el programa de manejo integrado de vectores durante la emergencia sanitaria generada por la COVID-19, así como hacer cambios en las estrategias de vigilancia entomológica que permitieron fortalecer la toma de decisiones tempranas para mitigar la ocurrencia de brotes de enfermedades transmitidas por vectores en la ciudad.
